# Identification of a New Sprouty Protein Responsible for the Inhibition of the *Bombyx mori* Nucleopolyhedrovirus Reproduction

**DOI:** 10.1371/journal.pone.0099200

**Published:** 2014-06-10

**Authors:** Shengkai Jin, Tingcai Cheng, Liang Jiang, Ping Lin, Qiong Yang, Yang Xiao, Takahiro Kusakabe, Qingyou Xia

**Affiliations:** 1 State Key Laboratory of Silkworm Genome Biology, Southwest University, Chongqing, China; 2 Sericulture and Farm Product Processing Research Institute, Guangdong Academy of Agricultural Sciences, Guangzhou, China; 3 Laboratory of Silkworm Science, Kyushu University Graduate School of Bioresource and Bioenvironmental Sciences, Fukuoka, Japan; Uppsala University, Sweden

## Abstract

The rat sarcoma-extracellular signal regulated kinase mitogen-activated protein kinases pathway, one of the most ancient signaling pathways, is crucial for the defense against *Bombyx mori* nucleopolyhedrovirus (BmNPV) infection. Sprouty (Spry) proteins can inhibit the activity of this pathway by receptor tyrosine kinases. We cloned and identified a new *B. mori* gene with a Spry domain similar to the Spry proteins of other organisms, such as fruitfly, mouse, human, chicken, Xenopus and zebrafish, and named it *BmSpry*. The gene expression analysis showed that *BmSpry* was transcribed in all of the examined tissues and in all developmental stages from embryo to adult. *BmSpry* also induced expression of BmNPV in the cells. Our results indicated: (1) the knock-down of *BmSpry* led to increased BmNPV replication and silkworm larvae mortality; (2) over-expression of *BmSpry* led to reduced BmNPV replication; and (3) *BmSpry* regulated the activation of ERK and inhibited BmNPV replication. These results showed that *BmSpry* plays a crucial role in the antiviral defense of the silkworm both *in vitro* and *in vivo*.

## Introduction

Sprouty (Spry) is a general inhibitor of receptor tyrosine kinases (RTKs), first identified in fibroblast growth factor (FGF)-stimulated tracheal branching during *Drosophila* development [1]. Multiple orthologues of *spry* have been reported in different organisms, including mouse, human, chicken, Xenopus and zebrafish [2]–[7]. The function of *spry* in the Lepidoptera, however, is not known. Mammalian genomes contain four *Spry* genes (*Spry 1*–*4*) encoding proteins (32–34 kDa) smaller than *Drosophila melanogaster* Spry (63 kDa) [8]. *Drosophila* Spry and vertebrate Spry proteins have a highly conserved C-terminal cysteine-rich region responsible for the membrane localization of Spry through palmitoylation [9]. A short region in the N terminus contains a conserved tyrosine residue, which mediates the interaction with its signaling molecules that contain Src-homology-2 domains [10]–[15].

Spry proteins are a major class of ligand-inducible inhibitors of RTK-dependent signaling pathways [16]–[17]. RTKs control a wide variety of processes, including proliferation, differentiation, migration and survival, in multicellular organisms [18]–[19]. In the RTKs- mitogen-activated protein kinase (MAPK) signaling pathway, the activated MAPKs phosphorylate and activate numerous target proteins, including transcription factors that regulate the expression of different genes [8], [20]–[22]. The results of earlier genetic experiments indicated that the inhibitory activity of Spry is upstream of the extracellular signal-regulated kinase (ERK) and downstream of the RTK [8]. Later studies suggested the precise point at which Spry intercepts RTK signaling varies depending on the biological context. Studies with *Drosophila* indicated that during eye development, Spry inhibits signaling downstream of the epidermal growth factor receptor (EGFR) and upstream of rous sarcoma (Ras) [1] but functions at the level of rapidly accelerated fibrosarcoma (Raf) during wing and ovary development [23].

RTKs-mediated signaling events must be regulated precisely both spatially and temporally to achieve refinement of an appropriate biological outcome [24]–[27]. A salient feature of the RTK signaling pathway is the transcriptional induction of negative regulators by the pathways that are eventually inhibited, thereby providing an effective mechanism for the coordination of signaling input with the physiological response [28]–[34]. One such negative regulator is Spry, a multifaceted negative-feedback repressor of RTK signaling in vertebrates and invertebrates [35]–[36]. Activation of RTK leads to the phospholipid-dependent translocation of Spry to the plasma membrane, where it is tyrosine phosphorylated by an Src-like kinase activity [35], [37]. Spry terminates this pathway by inhibiting the activation of Ras. And the study of Ras is well done in silkworm[38]–[42]. Unphosphorylated Spry might also block the Ras-ERK pathway by inhibiting Raf1 activation through an independent mechanism [12]. At the transcription level, activation of RTK leads also to the expression of *Spry* [43].

The available experimental evidence points to several mechanisms, all of which involve the interaction of Spry with essential elements of the RTK-Ras-ERK/MAPK cascade. The MAPK cascade, one of the most ancient and evolutionarily conserved signaling pathways, is important for many processes in immune responses [44]. MAPKs often have crucial roles in virus infection. Viral infection disorders the normal host cellular pathways, some of which reflect the organism's response to infection, whereas others are due to modification of the cellular environment by the virus [45]–[48]. For instance, modulation of MAPK pathways is essential for infection and replication of hepatitis B, Epstein–Barr, adenovirus and vaccinia viruses [49]–[52]. Further, it has been shown that activation of *B. mori* MAPKs BmERK and BmJNK are required for *B. mori* nucleopolyhedrovirus (BmNPV) infection in BmN cells [53].

We cloned and identified a homologue of *D. melanogaster Spry* from the B. mori genome, and named it *BmSpry*. Using RNAi and over-expression *in vivo* and *in vitro*, we proved that *BmSpry* has a function in antiviral defense through regulation of the activation of ERK. This is the first report that Spry protein is involved in the antivirus response in the Lepidoptera.

## Materials and Methods

### Silkworm strain, cell lines and viruses

*B. mori* DZ SN and Nm DZ lines were from the Gene Resource Library of Domesticated Silkworm (Southwest University, China). The BmE cell line[54] was cultured at 27°C in GRACE medium supplemented with 10% (v/v) fetal bovine serum (FBS). The BmN4-SID1 cell line was cultured at 27°C in IPL-41 medium supplemented with 10% (v/v) FBS [55]. BmNPV (Guangdong strain, China) and BmNPV-GFP were used in this study. Viruses were propagated in BmE cells and silkworm larvae, and BV titers were determined by plaque assay [56].

The mortality of DZ SN and Nm DZ lines after oral inoculation with wild type BmNPV *per os* of the newly exuviated 2nd or 4th instar larvae were measured as described [57]–[58].

### cDNA cloning, RT-PCR and qPCR analysis of *BmSpry*

Total RNA was prepared using the Total RNA Kit II (Promega) [59]. Total RNA (2 µg) was reverse transcribed as described [59]. For cDNA cloning, the GC buffer set (TaKaRa) and LA taq (TaKaRa) were used for PCR amplification with G+C-rich genes [60]–[61]. The primers used were: BmSpryF 5′-CGGTCGTTTCGTTGGAGC-3′ and BmSpryR 5′-ATTTGCGTAGAATCCAAAATACTAAC-3′. The nucleotide sequence reported in this paper has been submitted to the DDBJ/EMBL/GenBank Database under accession number KJ736835 (*BmSpry*). The qPCR/RT-PCR primers were: BmSpryF 5′-GCAGTGAATCCGCACGAGTT-3′ and BmSpryR 5′-CGCTGAAGTTCTTCGTTGATTTC-3′. The housekeeping gene BGIBMGA003186-TA was used as an internal control to standardize the variance among the different templates as described [57]–[58]. QPCR was done with the ABI StepOnePlus Real-Time PCR System and ABI 7500 Fast Real-Time PCR System (Applied Biosystems).

### Knockdown of *BmSpry* in BmE cells, BmN4-SID1 cells and in individuals

The dsRNAs for *BmSpry* and DsRed were generated by using a RiboMAX Large Scale RNA Production System-T7 kit (Promega) [62]. The primers were: T7-BmSpryF 5′-TAATACGACTCACTATAGGGGTGAAGGCGCTGTTCTACCA-3′, T7-BmSpryR (5′-TAATACGACTCACTATAGGGAGCGATCACTGAGTCCACCC-3′, T7-DsRedF 5′-TAATACGACTCACTATAGGGGTACGGCTCCAAGGTGTACG-3′, T7-DsRedR 5′-TAATACGACTCACTATAGGGGGTGTAGTCCTCGTTGTGGG-3′.

BmE cells (5×10^5^) were transfected with 4 µg of dsRNA using the TransMessenger™ Transfection Reagent (Qiagen) [63]. At 3 days post-transfection, cells were challenged with BmNPV-GFP at an MOI of 1. At 3 days after infection, cells were processed for immunofluorescence and harvested for qPCR [64].

BmN4-SID1 cells (5×10^5^) were used for RNAi by adding 500 ng of dsRNA directly into 1 ml of medium supplemented with 10% FBS [55]. After 5 days of RNAi, the rest of the process was as described above for the BmE cells.

Total DNA was obtained from the cells with the Universal Genomic DNA Extraction Kit Ver 3.0 (TaKaRa) as described [65]. The qPCR primer GP64 was used for analysis of BmNPV replication with primers: GP64F 5′-CCATCGTGGAGACGGACTA-3′, GP64R 5′-CTCGCACTGCTGCCTGA-3′, BmGAPDHF 5′-CATTCCGCGTCCCTGTTGCTAAT-3′, BmGAPDHR 5′-GCTGCCTCCTTGACCTTTTGC-3′. The housekeeping gene *BmGAPDH* was used as an internal control to standardize the variation among the different templates.

Nm DZ newly exuviated 5th instar larvae were injected with 30 µg of dsRNA [66]. Three days after RNAi, the larvae were injected with 2 µl of virus (10^6^ pfu/ml) by stab inoculation as described [67]. Total DNA was obtained at 3 days after infection. Each sample was extracted from five treated larvae and used for qPCR analysis of BmNPV replication as described [58].

### Over-expression of *BmSpry* in BmE cells

The *BmSpry* gene was amplified from the Nm DZ cDNA. The termination signal SV40 was cloned from the piggyBac [3×p3 EGFP afm] vector. The BmActin 4 (A4) promoter was used for the vector. The three elements were added to the empty vector 1180. BmE cells (5×10^5^) were transfected with 0.8 µg of [A4-BmSpry-SV40] vector using the X-tremeGENE HP DNA Transfection Reagent (Roche) [68]. At 2 days after transfection, cells were challenged with BmNPV-GFP at an MOI of 1. At 3 days after infection, cells were processed for immunofluorescence and harvested for qPCR as described [64].

### Western blotting

BmE cells (5×10^5^) were transfected with 0.8 µg of vector. At 2 days after transfection, cells were challenged with BmNPV-GFP at an MOI of 10. After infection for 24 h the cells were harvested for western blotting. RIPA lysis buffer was used to extract the total proteins within PhosSTOP (Roche) and PMSF (Roche) and 10 µg of total protein was used for western blotting analysis as described [53]. The antibody of anti-phospho-ERK (Cell Signaling Technology) and anti-GAPDH (Sigma Aldrich) were used in this experiment.

BmN4-SID1 cells (5×10^5^) were added to 500 ng of dsRNA in 1 ml of medium. After 5 days of RNAi, cells were challenged with BmNPV-GFP at an MOI of 10. After infection for 24 h, the cells were harvested for western blotting as described [53].

## Results

### Characteristics of the *BmSpry* sequence

**Figure 1:**
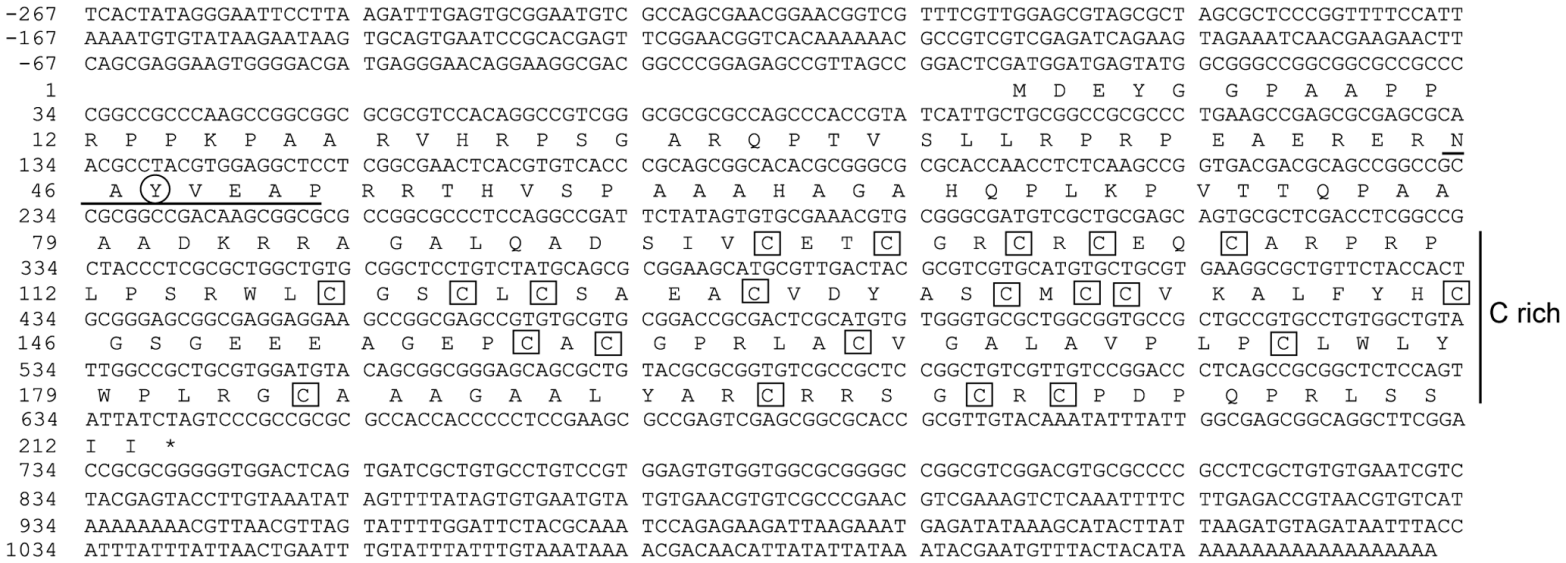
Characteristics of the *BmSpry* sequence. The nucleotide sequence is 1398-hand margin begins with the A of the presumptive initiating AUG codon. The 213 amino acid sequence is presented, terminated by an asterisk (*). The N terminus has a short conserved motif (underlined) and a conserved residue Y (indicated by a circle). A Cys-rich region (21 residues) is marked C rich at the right.

To analyze the features of the *BmSpry* sequence, we cloned this gene using LA taq and GC buffer. The *BmSpry* cDNA sequence contains 1398 nucleotides (Fig. 1) and the open reading frame, which consists of 642 nucleotides, is G+C-rich (71.2%). The sequence encodes a propeptide consisting of 213 amino acid residues, which has a calculated molecular mass of 22,717 Da. The predicted isoelectric point (pI) of the mature peptide is 8.90.

*BmSpry*, similar to *D. melanogaster Spry*, is a single-copy gene. The predicted protein sequence contains a Sprouty domain in its C terminus, which is conserved from invertebrates to vertebrates. This cysteine-rich domain consists of 104 amino acids (positions 100–203). Although the N terminus was more variable than the C terminus, the motif at amino acid positions 45–51 contained an invariant tyrosine residue (Y47) conserved between invertebrates and vertebrates (Fig. 1). Many of the inhibitory functions of Spry proteins are dependent on this tyrosine residue[43], [69].

### Expression pattern of *BmSpry*

**Figure 2:**
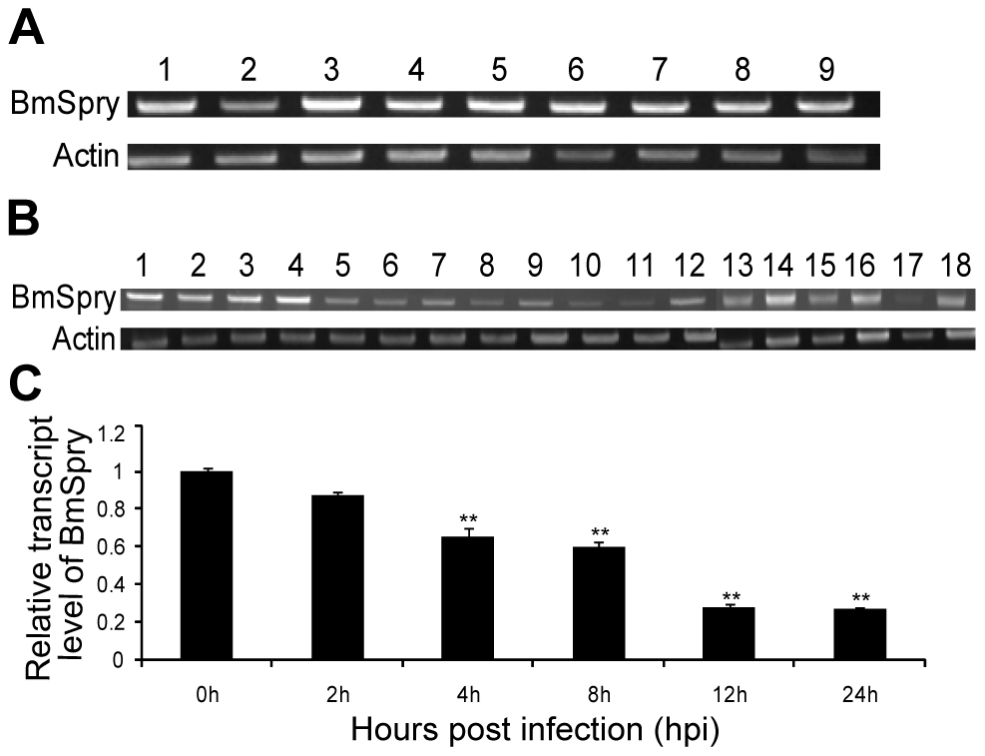
Expression profile of *BmSpry* in different tissues, developmental stages and inducible expression by BmNPV. (A) Expression of *BmSpry* in multiple tissues of 3^rd^-day 5^th^ instar larvae. Lanes: 1, head; 2, integument; 3, hemocyte; 4, malpighian tubule; 5, midgut; 6, fat body; 7, silk gland; 8, testis; and 9, ovary. (B) Expression of *BmSpry* at developmental stages from embryo to adult. Lanes: 1, 2 days after egg laying (AEL); 2, 4 days AEL; 3, 6 days AEL; 4, 8 days AEL; 5, newly hatched larvae; 6, molting larvae (ML) of the 1st instar; 7, newly exuviated larvae (NEL) of the 2nd instar; 8, ML of the 2nd instar; 9, NEL of the 3rd instar; 10, ML of the 3rd instar;11, NEL of the 4th instar; 12, ML of the 4th instar; 13, NEL of the 5th instar; 14, pupation in (PI) 2 days; 15, PI 4 days; 16, PI 6 days; 17, PI 8 days; and 18, adult moth. (C) Analysis of *BmSpry* gene expression in BmNPV-infected BmE cells at MOI of 10. Total RNA was extracted from the BmE cells at the indicated time points post-infection. Non-infected cells were used as the control and qPCR of *BmSpry* was done. A representative of triplicate experiments is shown. Data are given as mean ±SD (*n* = 3). Statistically significant differences: ** P<0.01.

The expression profile of *BmSpry* during the developmental stages from embryo to adult was investigated by RT-PCR using cDNAs from different tissues as templates. *BmSpry* was transcribed in all of the tissues examined (Fig. 2A) and the expression level of BmSpry was similar in all of these tissues, except the integument (Fig. 2A, lane 2). *BmSpry* was transcribed in all developmental stages from embryo to adult, but the expression levels were markedly higher in the egg, pupa, 4th and 5th instar compared to the other stages of development (Fig. 2B).

Next, we used time-course analysis in the BmE cells by quantitative PCR (qPCR) to analyze the induced expression level of *BmSpry* after BmNPV infection (Fig. 2C). We chose five post-infection time points as the experimental group and time zero as a negative control. The results indicated the *BmSpry* expression level was down-regulated gradually from 2–24 h and was down-regulated markedly by BmNPV infection at 12 h and 24 h. These results implied *BmSpry* might have a function in the process of BmNPV infection.

### Restricting BmNPV infection in cultured cells

**Figure 3:**
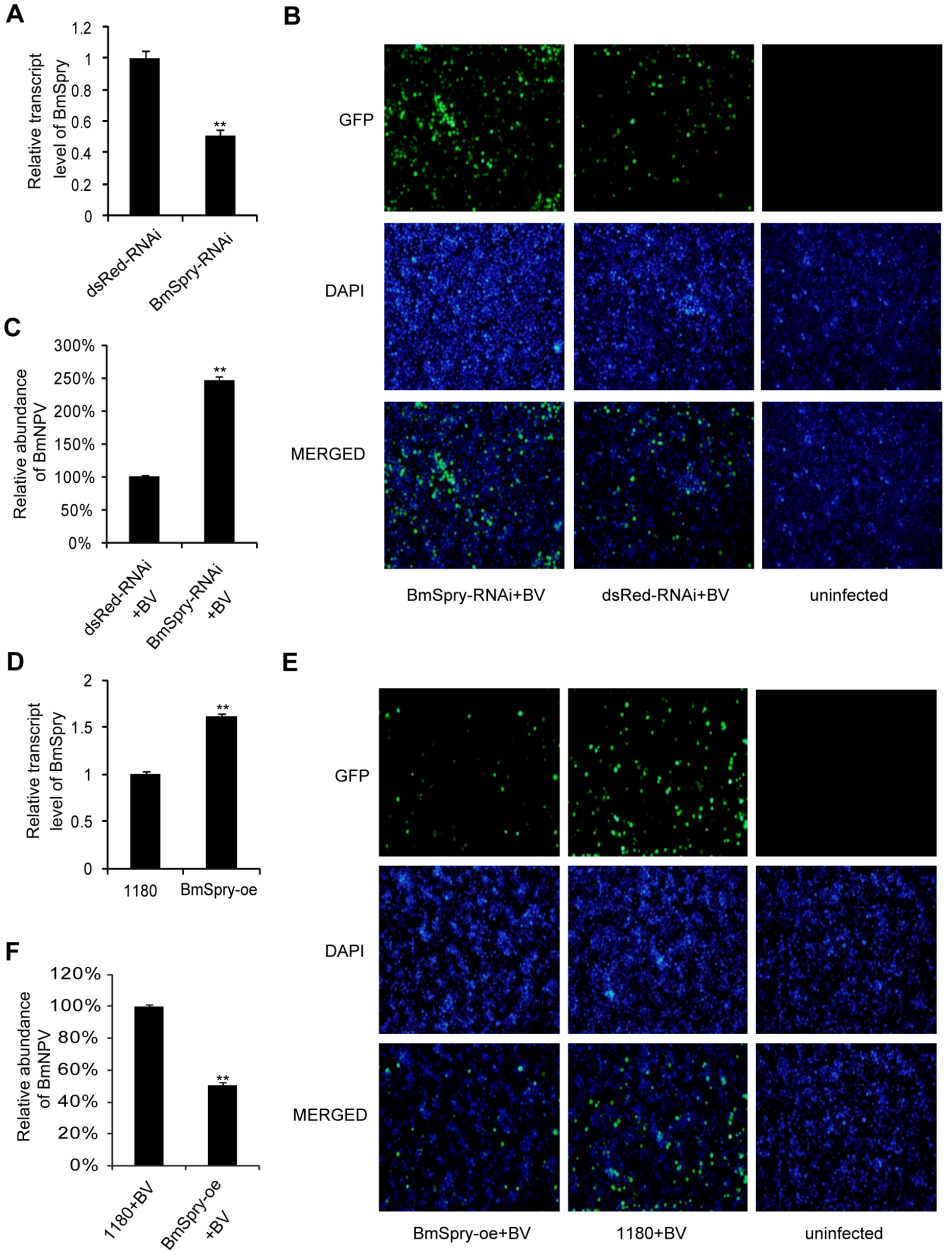
*BmSpry* inhibited BmNPV replication in cultured cells. (A) BmE cells pretreated with dsRNA as indicated, the dsRNA of dsRed was used as a negative control. At 3 days post transfection, total RNA was extracted and qPCR was used to analyze the *BmSpry* expression level. (B) BmE cells treated with dsRNA against the indicated genes were infected with BmNPV-GFP at MOI of 1 for 3 days and processed for immunofluorescence. (C) BmE cells treated with the indicated dsRNA were infected with BmNPV-GFP at MOI of 1 and infection total genomes were extracted for qPCR at 3 days post. (D) BmE cells were used for transient transfection and the empty vector 1180 was used as a negative control. BmSpry-oe was an over-expression vector of BmSpry. At 3 days post-transfection, total RNA was extracted for qPCR. (E) BmE cells were subjected to transient transfection with *BmSpry* expression vectors and the empty 1180 vector, as indicated. At 2 days post-transfection, the cells were infected with BmNPV-GFP at MOI of 1 for 3 days and then processed for immunofluorescence. (F) BmE cells transfected with the indicated vectors were infected with BmNPV at MOI of 1 and total genomes were extracted for qPCR at 3 days post-infection. A representative of triplicate experiments is shown. Data are given as mean ±SD (*n* = 3). Statistically significant differences: ** P<0.01.

To determine whether BmSpry was involved in antiviral defense against BmNPV, we generated the double-stranded RNA (dsRNA) of *BmSpry* and repressed *BmSpry* in BmE cells via RNA interference (RNAi). The results showed silencing for *BmSpry* was efficient and the expression level of *BmSpry* was markedly reduced (Fig. 3A). Next, we challenged RNAi-treated cells with BmNPV-green fluorescent protein (GFP) and then investigated the BmE cells by fluorescence microscopy at 3 days post-infection. The result showed a significant increase of infected cells upon silencing *BmSpry* but not the *Discosoma* red fluorescent protein (dsRed) (Fig. 3B). We extracted the total genomes of the infected cells for qPCR analysis and the results indicated there was a significant increase in the amount of viruses of about twofold compared to the BmE cells when depleting *BmSpry* by RNAi (Fig. 3C).

We used BmN4-SID1 cells to repeat the RNAi experiment for further verification of the RNAi results and obtained the same results as those found for the BmE cells. Firstly, we confirmed the BmN4-SID1 cells reduced the *BmSpry* expression level significantly without the use of a transfection reagent (Fig. S1A). Then, we challenged RNAi-treated BmN4-SID1 cells with BmNPV and analyzed the amount of viruses, as we did with BmE cells. The results showed the amount of viruses in the BmSpry-depleted cells was more than twofold greater compared to the control cells (Fig. S1B).

We up-regulated the expression level of *BmSpry* in the BmE cells by transient transfection and over-expression of *BmSpry* was verified by qPCR (Fig. 3D). At 48 h post transfection, we challenged over-expression-treated cells with BmNPV-GFP and then analyzed the infection by fluorescence microscopy. The percentage of infected cells was notably reduced in the cells with over-expression of *BmSpry* (Fig. 3E). Furthermore, qPCR analysis confirmed over-expression of *BmSpry* inhibited BmNPV replication and reduced the amount of viruses to half (Fig.3F). Taken together, our data suggest that *BmSpry* has an important role in BmNPV infection *in vitro*.

### BmSpry inhibits BmNPV replication through regulation of ERK activation

**Figure 4:**
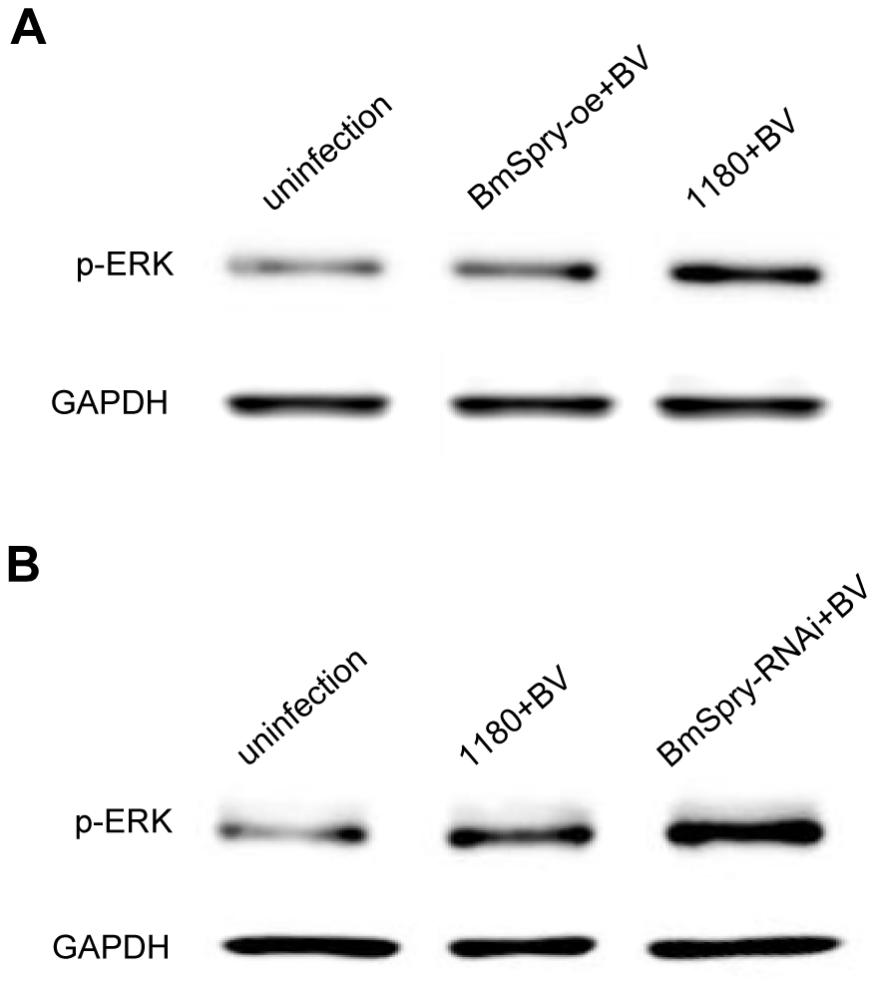
BmSpry regulation of ERK activation inhibited BmNPV replication. (A) Firstly, BmE cells were transfected. The empty vector 1180 was used as a negative control and the BmSpry-oe vector was used as the experimental group with over-expression of *BmSpry*. At 2 days post-transfection, BmE cells were infected with BmNPV at MOI of 10. Post infection for 24 h, total proteins were extracted for western blotting using anti-phospho-ERK antibody. Total protein levels were measured with anti-GAPDH antibody. (B) BmN4-SID1 cells were used. After RNAi of 5 days, the BmN4-SID1 cells were infected with BmNPV at MOI of 10. Post infection for 24 h, total proteins were extracted for western blotting. A representative of triplicate experiments is shown.

Early work showed activation of ERK and JNK is required for BmNPV infection in cultured cells[53]. In this study, we used over-expression and RNAi of *BmSpry* in BmNPV-infected cells to investigate whether the antiviral defense function of *BmSpry* acts through inhibition of ERK activation. ERK had a basic level of phosphorylation in uninfected cells. When the cells had been infected with BmNPV for 24 h, however, the activation of ERK was enhanced significantly. Interestingly, the activation state of ERK induced by BmNPV infection can be inhibited markedly in BmE cells by over-expression of *BmSpry* (Fig. 4A). Furthermore, down-regulation of *BmSpry* in BmN4-SID1 cells can enhance the activation state of ERK significantly by BmNPV infection (Fig. 4B). These results suggested regulation of the ERK pathway by the BmSpry protein inhibited replication of the virus.

### Antiviral defense *in vivo*

**Figure 5:**
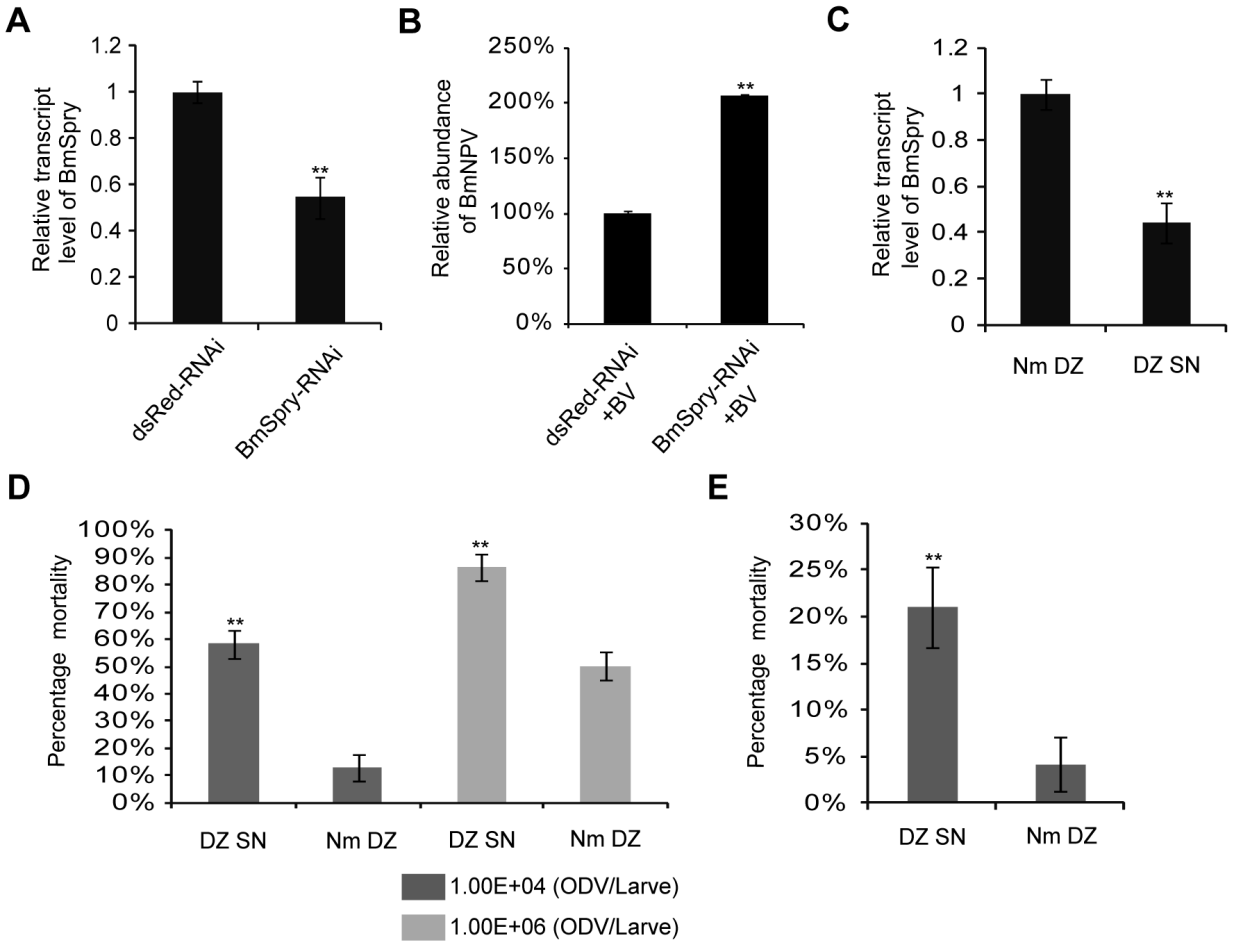
*BmSpry* was essential for antiviral defense *in vivo*. (A) The silkworm Nm DZ line was used for the RNAi experiment. Injection of dsRNA is indicated, the dsRNA of dsRed was used as a negative control with dsRNA of *BmSpry* as the experimental group. At 3 days post-injection of dsRNA, total RNA was extracted for qPCR to measure the expression level of *BmSpry*. Data are given as mean ±SD (*n* = 3). (B) Nm DZ silkworms treated with dsRNA against the indicated genes were infected with BmNPV (10^6^ pfu/mL) by stab inoculation for 3 days and processed for qPCR. Data are given as mean ±SD (*n* = 3). (C) Analysis of the expression level of *BmSpry* in Nm DZ and the mutant DZ SN. Total RNA extracted from newly exuviated Nm DZ and DZ SN 5th instar larvae was used for qPCR. Data are given as mean ±SD (*n* = 3). (D) Analysis of mortality of DZ SN and Nm DZ after oral inoculation with BmNPV. Nm DZ and DZ SN newly exuviated 4th instar larvae were used for this experiment. DZ SN and Nm DZ silkworms were infected with BmNPV *per os* using a dose of 10^4^ or 10^6^ OB/larva and mortality was monitored until the adult stage (22 days). (E) Newly exuviated 2nd-instar larvae were used to investigate mortality after inoculation *per os* with 5×10^3^ OB/larva. Mortality was monitored until the pupa stage (16 days). A representative of triplicate experiments is shown. Statistically significant differences: ** P<0.01.

We showed *BmSpry* was antiviral *in vitro* and asked whether it had similar biological function *in vivo*. Using RNAi *in vivo*, we investigated whether loss of *BmSpry* had an effect on BmNPV replication. Knock-down *BmSpry* in silkworm larvae was generated by injection of 30 µg of dsRNA against *BmSpry*. Efficient silencing was confirmed by qPCR; the expression level of *BmSpry* was reduced by 40% (Fig.5A). Subsequently, we challenged RNAi-treated larvae with BmNPV by stab inoculation and analyzed viral DNA replication with qPCR. There was a significant increase, about twofold, of viral DNA replication upon silencing of *BmSpry* but not the dsRed (Fig. 5B).

Next, we used silkworm lines DZ SN and Nm DZ to investigate the mortality following infection with BmNPV *per os*. The Nm DZ line was the wild type and the DZ SN line had the same genetic background apart from a transposon insertion 14.6 kb upstream of *BmSpry*. Interestingly, the expression level of *BmSpry* was markedly lower in DZ SN compared to Nm DZ (Fig. 5C). Newly exuviated 4th-instar larvae of the two lines were infected with BmNPV *per os* using a dose of 10^4^ or 10^6^ occlusion bodies (OB)/larva. The results showed mortality was significantly higher in the DZ SN line compared to the Nm DZ line for each dose of OB. At the lower dose, mortality was nearly 10% in the Nm DZ line and reached 60% in the DZ SN line. At the higher dose, mortality was 50% in the Nm DZ line and 90% in the DZ SN line (Fig. 5D).

The same results for mortality were found in newly exuviated 2nd-instar larvae following infection with BmNPV *per os* at a dose of 5×10^3^ OB/larva. The Nm DZ line had a mortality of ∼5% and the DZ SN line had a mortality >20% (Fig. 5E). Overall, these results showed the DZ SN line was significantly susceptible to BmNPV, suggesting *BmSpry* has an important antiviral role in *vivo*.

## Discussion

The essential roles for two MAPKs, ERK and JNK, in the efficient infection of silkworm cells was firmly established [53]. It was unclear, however, how silkworm larvae resist virus infection using these signaling pathways and how viruses overcome the host resistance. In this study, we cloned a gene and named it *BmSpry*. Expression pattern analysis showed it was transcribed in all larval tissues and all developmental stages from egg to adult moth. We showed also *BmSpry* had the ability to normalize the activation of ERK induced by BmNPV infection and the virus had the ability to down-regulate *BmSpry*, rendering the MAPK signaling pathway out of control. This is the first report of this important function of *BmSpry* in antiviral defense.

*Spry*, a general inhibitor of RTKs [16]–[17], has many copies in some organisms; e.g. four copies in the human and mouse genomes [8]. There is only one copy in the silkworm, however, which is likely why *BmSpry* was expressed in all of the larval tissues and at all developmental stages[70]–[72]. The expression level of *BmSpry* was very similar among all tissues, suggesting *BmSpry* has the same function in all silkworm tissues. At different developmental stages, however, there was a great diversity of expression level, with especially high levels in the egg and pupa. This result implies *BmSpry* has a crucial role in development of the individual. The *spry* gene must regulate RTK-mediated signaling pathways precisely to ensure the appropriate biological outcome.

It is proven that *spry* is a general inhibitor of RTKs [16]–[17] and each RTK signaling pathway has a salient feature requiring exact feedback regulation systems [28]–[34]. In a normal individual, activation of ERK is followed by translocation to the nucleus and induction of *spry* transcription [43]. Feedback of the newly synthetized spry protein regulates the MAPK signaling pathway to the normal level. In the silkworm, stable expression of *BmSpry* ensures the appropriate biological outcome. When the virus disequilibrates the RTK-MAPK signaling pathway and enhances the level of ERK activation, *BmSpry* acts as an RTK signaling pathway inhibitor restoring the balance and restricts the use of RTKs by BmNPV for replication.

Baculovirus–host interactions are a hot topic for research but progress is slow. Since it was found baculovirus use the MAPK signaling pathways for replication [53], the search for the receptor of the upstream of MAPK has never been abandoned. In this study, we showed *BmSpry* is involved in the MAPK signaling pathway, indicating the receptor of the upstream of MAPK belongs to the RTK family. This finding allows the search for the receptor to be more explicit and specific. Further, we suggest the baculovirus uses the host growth factors to activate the RTKs because the BmNPV genome contains only one of the growth factors, vFGF [73], and is not necessary for BmNPV to activate MAPK [53]. In the BmNPV-infected cells, the virus could activate the ERK and down-regulate *BmSpry* simultaneously. This infection mechanism allows the virus unlimited use of the cell resources for maximum replication.

In conclusion, our results show that *BmSpry* is involved in BmNPV infection. *BmSpry* is able to act against baculoviruses, modulating the MAPK signaling pathways and normalizing the superactivation of ERK by virus infection. The results of our study contribute to elucidation of the mechanism that allows baculovirus to modulate the MAPKs and confirm RTKs are upstream of MAPKs during baculovirus infection.

## Supporting Information

Figure S1 *BmSpry* inhibited BmNPV replication in BmN4-SID1 cells. (A) The BmN4-SID1 cells were used for the RNAi experiment and the dsRNA of dsRed was used as a negative control. After 5 days of RNAi, total RNA was extracted for qPCR. (B) BmN4-SID1 cells treated with the indicated dsRNA were infected at MOI of 1 and the total genomes were extracted for qPCR at 3 days post-infection. A representative of triplicate experiments is shown. Data are given as mean ±SD (n = 3). Statistically significant differences: ** P<0.01.
(TIF)
