# Supplementary figures and images for: Identification of a New Sprouty Protein Responsible for the Inhibition of the Bombyx mori Nucleopolyhedrovirus Reproduction
Source: PLoS One. 2014 Jun 10;9(6):e99200. doi: 10.1371/journal.pone.0099200 (PMC4051654; doi:10.1371/journal.pone.0099200)

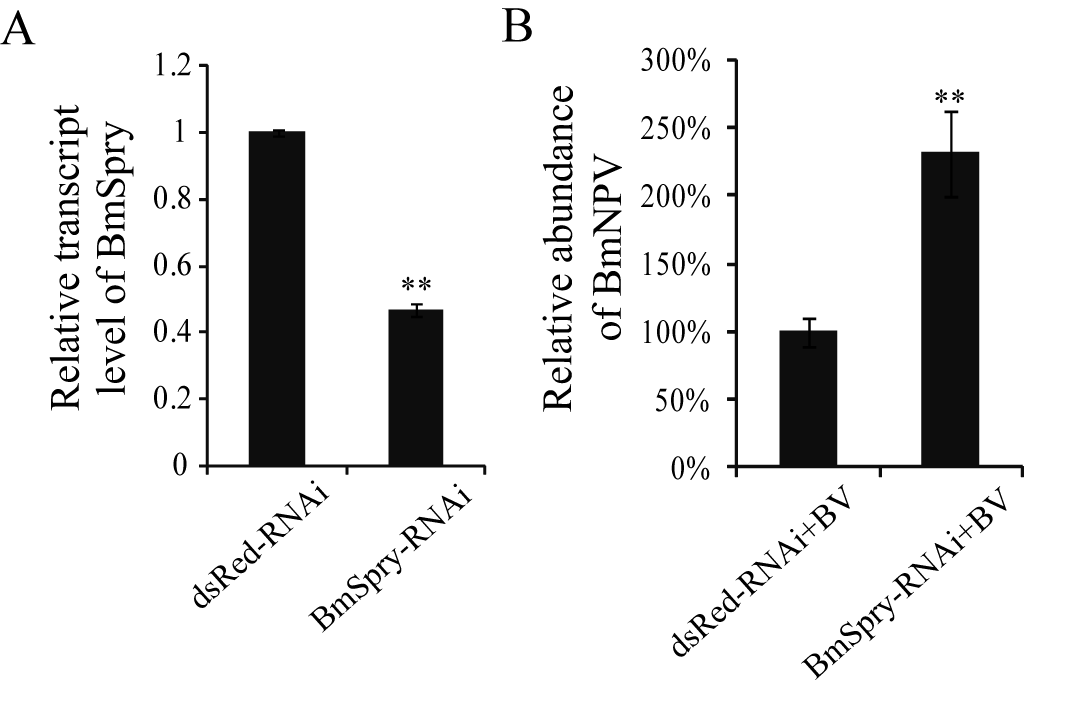

Supplement: Figure S1 — BmSpry inhibited BmNPV replication in BmN4-SID1 cells. (A) The BmN4-SID1 cells were used for the RNAi experiment and the dsRNA of dsRed was used as a negative control. After 5 days of RNAi, total RNA was extracted for qPCR. (B) BmN4-SID1 cells treated with the indicated dsRNA were infected at MOI of 1 and the total genomes were extracted for qPCR at 3 days post-infection. A representative of triplicate experiments is shown. Data are given as mean ±SD (n = 3). Statistically significant differences: ** P<0.01. (TIF) [file pone.0099200.s001.tif]
